# Supplementary material for: DKK3, Downregulated in Invasive Epithelial Ovarian Cancer, Is Associated with Chemoresistance and Enhanced Paclitaxel Susceptibility via Inhibition of the β-Catenin-P-Glycoprotein Signaling Pathway
Source: Cancers (Basel). 2022 Feb 12;14(4):924. doi: 10.3390/cancers14040924 (PMC8870560; doi:10.3390/cancers14040924)
Supplement: Supplementary file 1 [file cancers-14-00924-s001.zip › cancers-1552743-supplementary.pdf]

Supplementary materials: DKK3, Downregulated in Invasive Epithelial Ovarian Cancer, Is Associated with Chemoresistance and Enhanced Paclitaxel Susceptibility Via Inhibition of the β-Catenin-P-Glycoprotein Signaling Pathway

Que Thanh Thanh Nguyen, Hwang Shin Park, Tae Jin Lee, Kyung-Mi Choi, Joong Yull Park, Daehan Kim, Jae Hyung Kim, Junsoo Park and Eun-Ju Lee

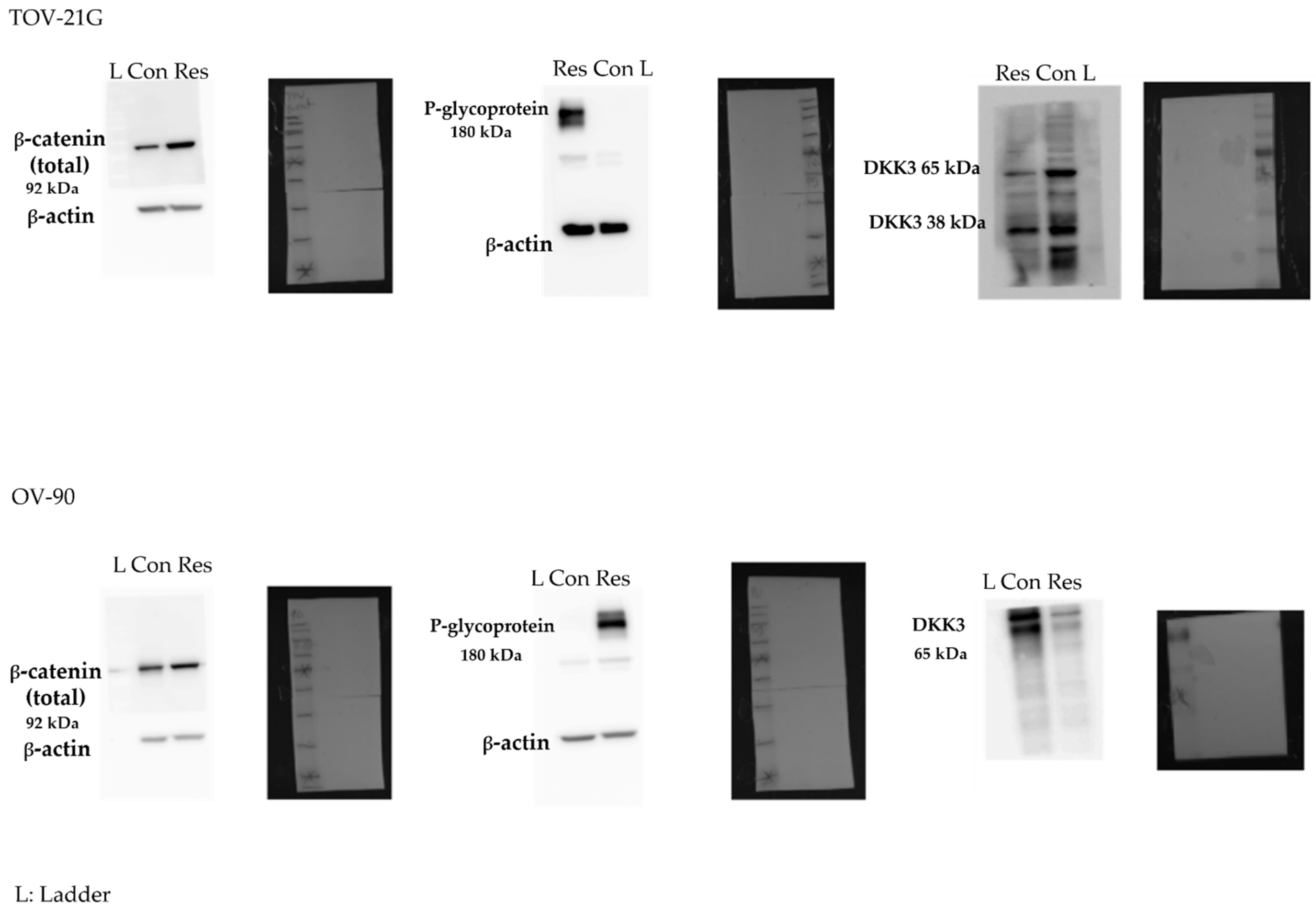

Figure S1. The original Western blots figures for Figure 2e.

Sup. 3

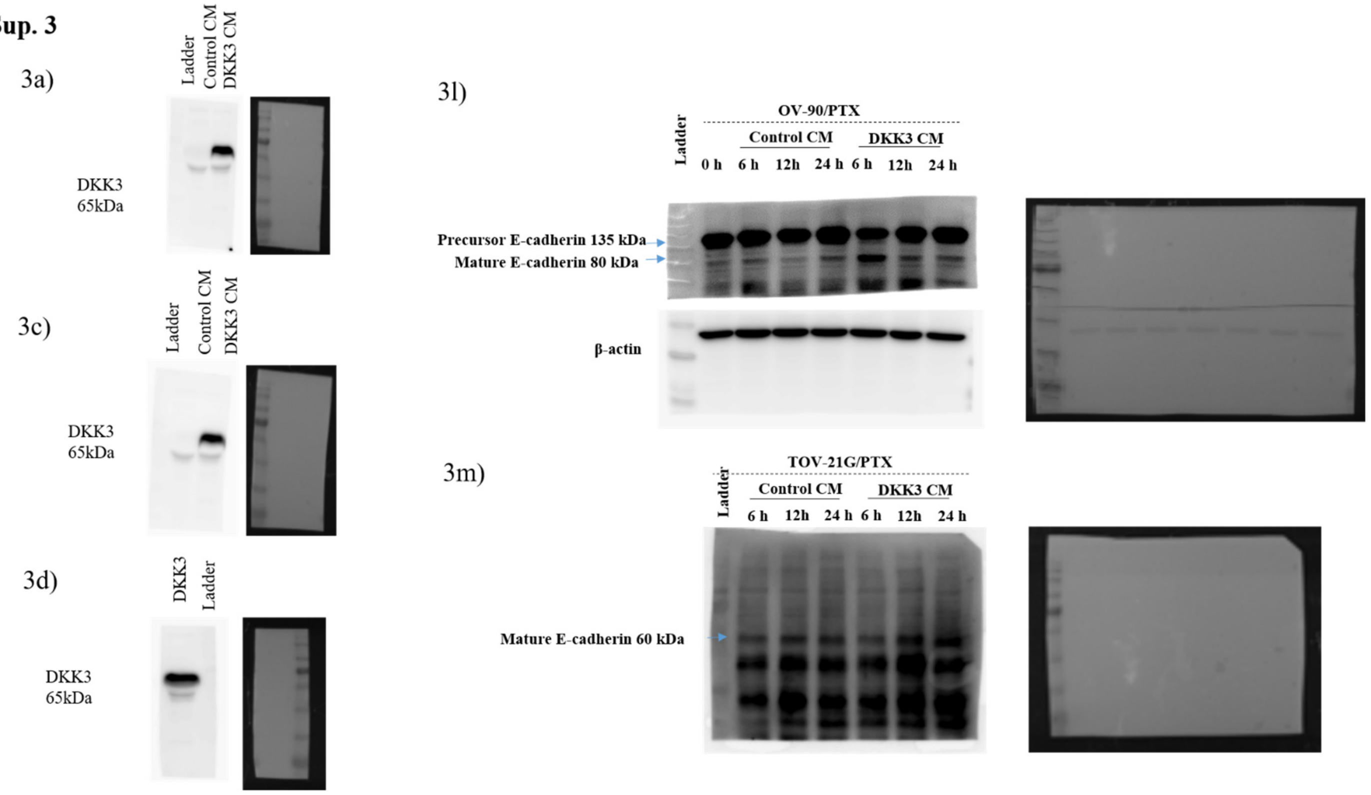

Figure S2. The original Western blots figures for Figure 3.

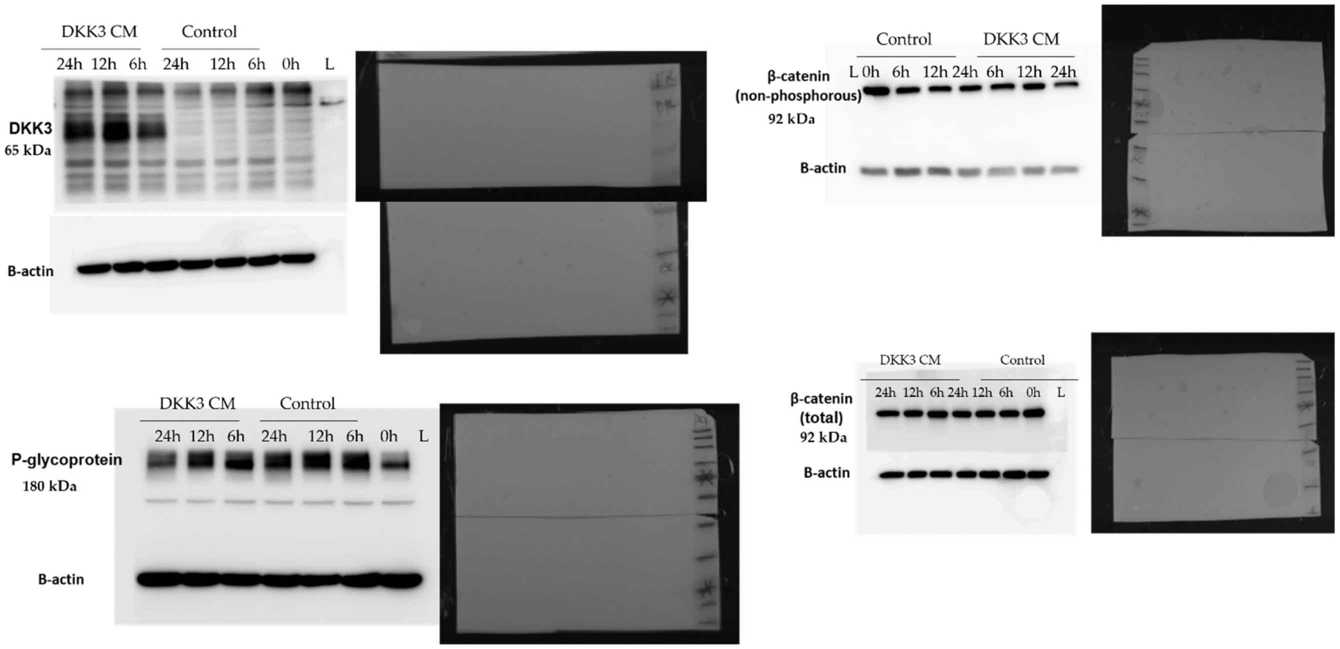

L: :Ladder

Figure S3. The original Western blots figures for Figure 4c.

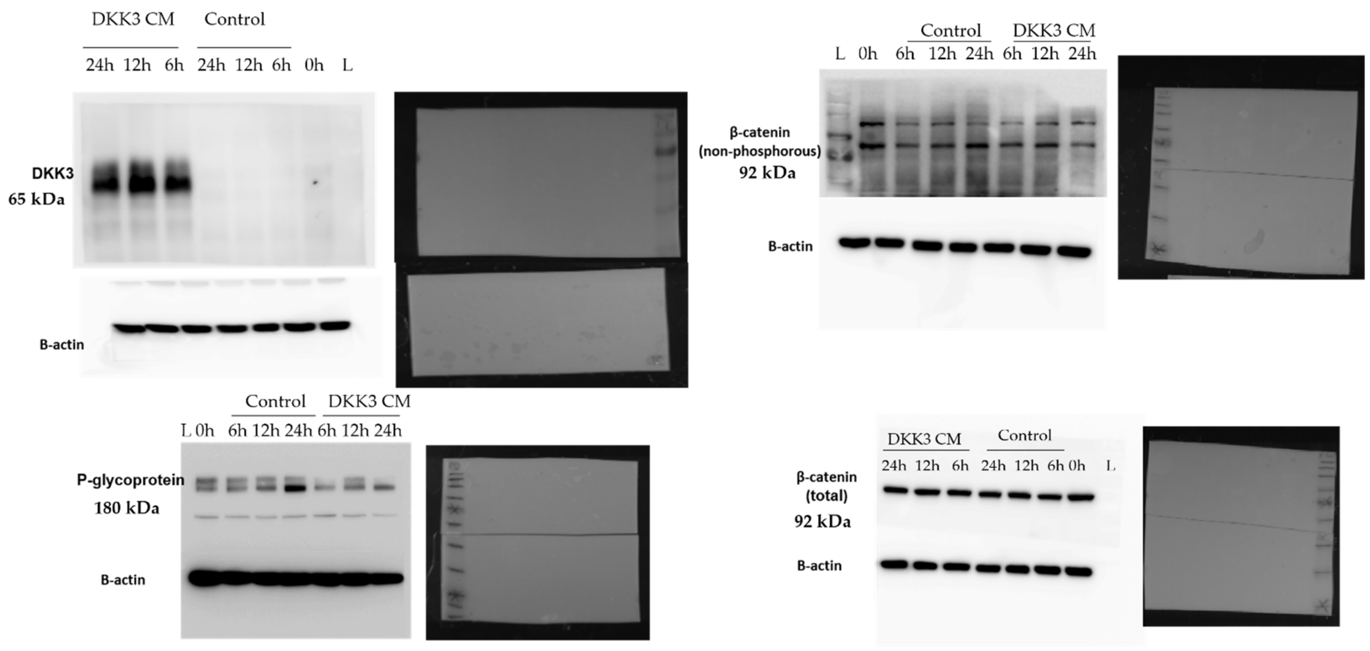

L: :Ladder

Figure S4. The original Western blots figures for Figure 4d.

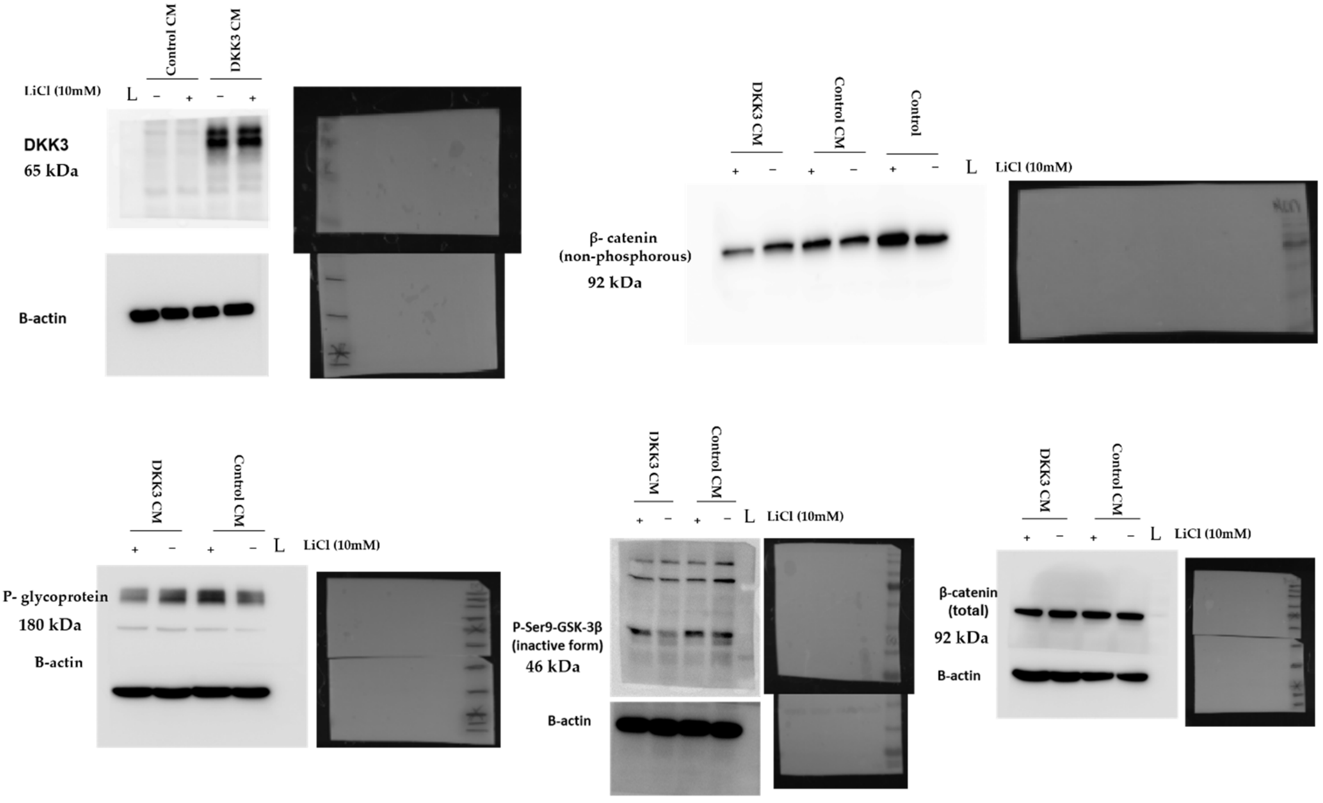

L: :Ladder

Figure S5. The original Western blots figures for Figure 4e.

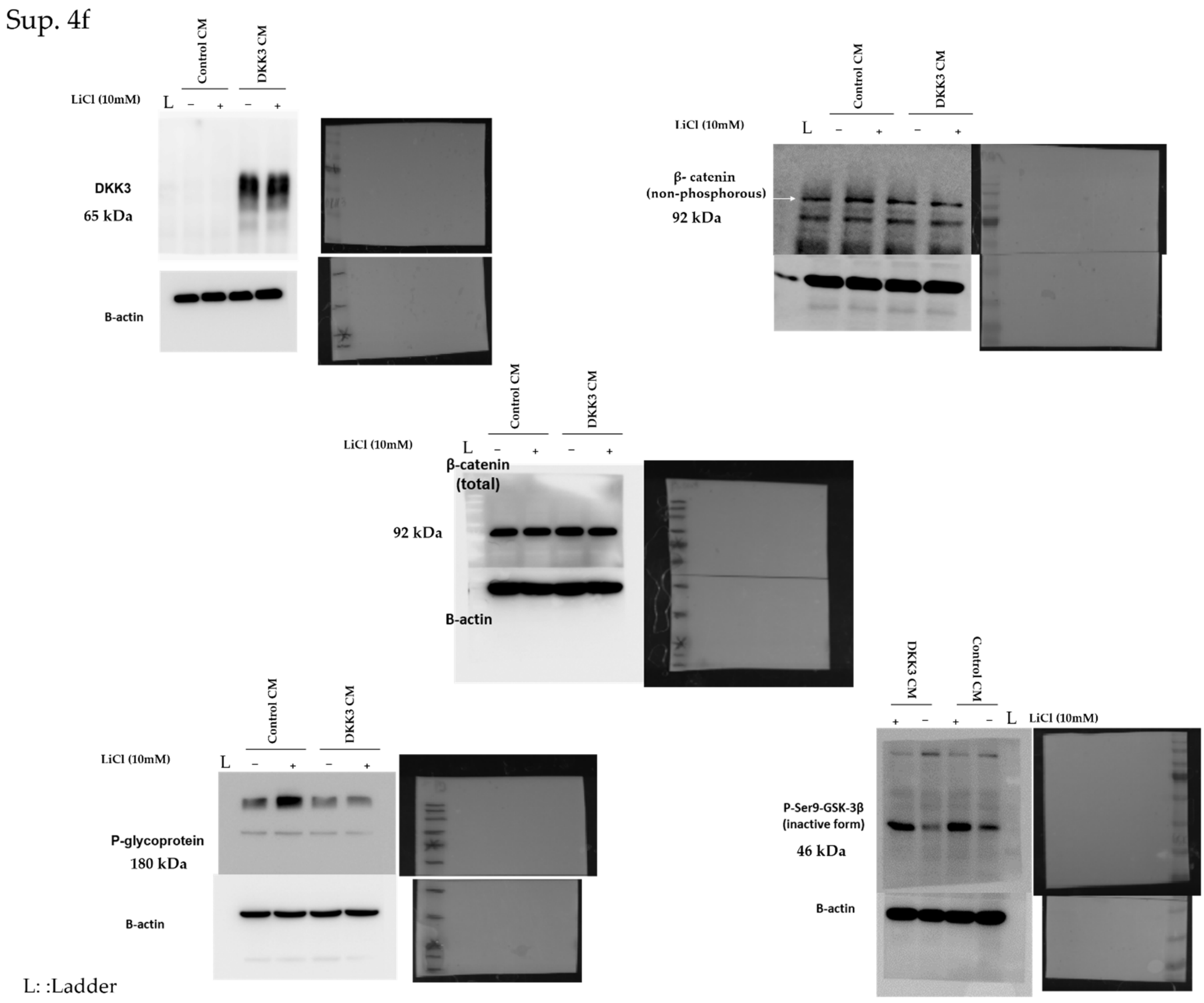

Figure S6. The original Western blots figures for Figure 4f.

Table S1. Clinicopathological parameters and disease-free survival analysis of prognostic factors in 82 patients.

**Clinicopathological Parameters**	***N* (%)**	***p*-Value**	
		**Univariate**	**Multivariate**
Age			
Mean (range), year	52.2 (24–78)		
CA125			
≤ 35 U/mL	14 (17.1)	0.026	NS
> 35 U/mL	68 (82.9)		
Histology			
Mucinous	13 (15.9)	NS	NS
Serous	42 (51.2)		
Endometrioid	5 (6.1)		
Transitional cell	12 (14.6)		
Clear cell	3 (3.7)		
Undifferentiated	7 (8.5)		
FIGO stage			

I-II	28 (34.1)	0.009	NS
III-IV	54 (65.9)		
DKK3 protein expression			
Negative	46 (56.1)	NS	NS
Positive	36 (43.9)		
Debulking operation			
Optimal	55 (67.1)	0.040	NS
Suboptimal	27 (32.9)		
Chemo-response			
Sensitive	33 (40.2)	<0.001	0.006
Resistant	21 (25.6)		
Unknown	28 (34.2)		

[*]NS, no significance.

Table S2. Clinicopathological characteristics of women with/without expression of DKK3.

**Clinicopathological Parameters**	**DKK3 Expression**		
	**Negative (*n* = 46)**	**Positive (*n* = 36)**	***p*-Value**
Age			[*]NS
Mean (±SD), year	51.9 (±12.2)	52.8 (±14.4)	
CA125			NS
≤ 35 U/mL	7	7	
> 35 U/mL	39	29	
FIGO stage			NS
I-II	10	18	
III-IV	36	18	
Debulking operation			NS
Optimal	29	26	
Suboptimal	17	10	
Chemo-response			NS
Sensitive	23	10	
Resistant	12	9	
Unknown	11	17	
Recurrence			NS
No	22	14	
Yes	21	22	
Unknown	3	0	

[*]NS, no significance.
